# Potential Role of Febrile Seizures and Other Risk Factors Associated With Sudden Deaths in Children

**DOI:** 10.1001/jamanetworkopen.2019.2739

**Published:** 2019-04-26

**Authors:** Laura Gould Crandall, Joyce H. Lee, Rebecca Stainman, Daniel Friedman, Orrin Devinsky

**Affiliations:** 1 Sudden Unexplained Death In Childhood Foundation, Roseland, New Jersey; 2 New York University School of Medicine, Comprehensive Epilepsy Center, New York

## Abstract

**Importance:**

Sudden unexplained death in childhood (SUDC) is the fifth leading category of death among toddlers but remains underrecognized and inadequately studied.

**Objective:**

To assess the potential role of febrile seizures (FS) and other risk factors associated with SUDC and describe the epidemiology, mechanisms, and prevention of SUDC.

**Design, Setting, and Participants:**

This case series study reviewed 622 consecutive sudden child death cases aged 1 to 17 years from 2001 to 2017 from 18 countries. Data were collected from family members of children who died suddenly; these families voluntarily registered with the SUDC Foundation. Data analysis was conducted from November 2017 to February 2019.

**Main Outcome Measures:**

Certified manner of death characterized as accident, natural, or undetermined.

**Results:**

A total of 391 families with decedents aged 1 to 6 years completed a comprehensive interview on medical and social histories, and circumstances of death with forensic evaluations revealing a cause of death (sudden explained death in childhood [SEDC]) or no cause of death (SUDC). Of these children, 231 (59.1%) were male, the mean (SD) age at death was 24.9 (12.8) months, and 104 (26.6%) had a history of FS. Compared with the general population FS prevalence (2%-5%), FS prevalence among SUDC (28.8%; 95% CI, 23.3%-34.2%) and SEDC (22.1%; 95% CI, 14.8%-29.3%) were elevated. The odds of death during sleep was 4.6-fold higher in SUDC than in SEDC cases (odds ratio, 4.61; 95% CI, 1.92-11.09; adjusted *P* = .008). The siblings of SUDC cases were followed up for 3144 life-years, and none died prematurely from SUDC.

**Conclusions and Relevance:**

This analysis of the largest SUDC cohort confirmed an increased FS rate and found significantly increased rates of FS among SEDC. This study suggests that seizures may contribute to some SUDC and SEDC deaths. The risk of sudden death in a sibling was low. To develop and assess preventive strategies, population-based studies are needed to define the epidemiology and spectrum of risk factors and identify biomarkers of patients with FS at high risk of sudden death.

## Key Points

**Question:**

Are febrile seizures associated with increased risk of sudden deaths in young children, and could febrile seizures contribute to some deaths?

**Findings:**

In this case series of 391 children from 18 countries, febrile seizure rates were increased among both sudden explained and sudden unexplained deaths compared with the general population, suggesting that seizures contributed to some of these deaths. No sudden deaths occurred in more than 3100 life-years among siblings of children with sudden unexplained death.

**Meaning:**

Patients with febrile seizures are at increased risk for sudden death, but the risk is small; identifying clinical features or biomarkers of high risk is essential to develop and assess preventive strategies.

## Teaser

This case series study assesses the potential role of febrile seizures and other risk factors associated with sudden death in childhood and describes the epidemiology, mechanisms, and prevention of sudden death in childhood.

## Introduction

Sudden unexplained deaths in children (SUDC) aged 12 months and older have largely escaped recognition by medical and research communities,^1,2^ with fewer than 20 SUDC-focused publications in the US National Library of Medicine through February 2019.^3^ In contrast, the epidemiology and risk factors for sudden infant death syndrome ([SIDS]; aged <1 year) are well defined.^4^ In the United States, 389 children aged 1 to 18 years died without explanation in 2017; 62.5% of these deaths were in children aged 1 to 4 years, the fifth leading category of death for this group.^5^ Some unexplained deaths are misattributed to a nonlethal pathology such as a mild upper respiratory tract infection, underrepresenting the SUDC incidence.^6^

Sudden unexplained death in childhood cases and their family members have elevated rates of febrile seizures (FS).^1,7,8,9,10,11^ We previously reported 123 cases from our current cohort^7^; 31.7% had 1 or more FS. Since more than 75% of SUDC cases had a terminal fever or illness symptoms within 48 hours of death, we postulated that in some cases, an FS may have contributed to or caused death.^7^ This is analogous to sudden unexpected death in epilepsy (SUDEP). However, since FS are considered provoked seizures (ie, not epilepsy), even if a terminal FS is witnessed, the cases are not classified as SUDEP.^7,12^ To more accurately define the prevalence of FS, identify other risk factors in SUDC cases, and compare these cases with sudden explained deaths in childhood (SEDC), we studied a large cohort of these children.

## Methods

To our knowledge, the SUDC Foundation is the only nonprofit organization whose purpose is to promote awareness, advocate for research, and provide bereavement support to those affected by SUDC and SEDC. Between 2001 to 2017, we enrolled 622 families from the United States (81.6%) and 17 other countries (Australia, Belgium, Brazil, Canada, Costa Rica, England, France, Germany, Iceland, Ireland, Italy, Namibia, New Zealand, Scotland, Singapore, South Africa, and Sweden). All cases had data on demographics and cause of death. Among the 622 cases, we studied cases who completed a comprehensive interview on the decedents’ and their families’ social and medical histories and had a death investigation inclusive of an autopsy. We focused on the 391 children who died between ages 1 to 6 years, since this age group composed 94.4% of those interviewed.

Study inclusion required that the decedent’s family voluntarily contacted the SUDC Foundation and that the initial intake confirmed that the child’s death was sudden, unexpected, and not the result of a known disease (eg, cancer, neurodegenerative disorder); the death was investigated and included an autopsy; and there was no active criminal investigation. The SUDC Foundation interview assessed the decedent’s birth and medical history, mother’s pregnancy, environmental factors, family medical history, circumstances of the child’s death, and final cause of death certification (eTable 1 in the Supplement). Final cause of death led to categorization as explained (SEDC) or unexplained (SUDC). Sudden explained deaths in childhood included natural infectious, natural noninfectious, or accidental deaths. Sudden unexplained deaths in children deaths were categorized as with FS, afebrile seizures, or no seizures (Figure). No independent medical record or autopsy review was performed. Parents consented to release deidentified data for research. The foundation provided deidentified data for analysis. The New York University institutional review board considered this study exempt. Reporting is consistent with the Strengthening the Reporting of Observational Studies in Epidemiology (STROBE) reporting guideline.

**Figure.**
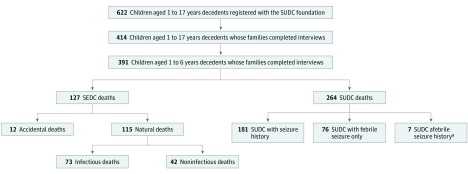
Categorization of Data Set SEDC indicates sudden explained death in childhood; and SUDC, sudden unexplained death in childhood. ^a^One decedent also had a history of simple febrile seizures.

### Statistical Analysis

Results were analyzed using 2-tailed *t* test for continuous variables and χ^2^ test of independence for categorical variables; *P* values were adjusted using the Holm-Bonferroni correction for multiple comparisons, denoted as adjusted *P* value.^13^ Odds ratios (ORs) with 95% CIs were calculated for significant associations between categorical variables.

## Results

Among the 622 children, 208 (33.4%) were SEDC and 414 (66.6%) were SUDC. Male sex and white race were more common in both SEDC and SUDC. Age at SEDC was younger than age at SUDC (median, 23 months; interquartile range [IQR], 17-37 vs median, 21 months; IQR, 17-29; adjusted *P* = .02). Seasonal variation of death was nearly identical between groups and highest in winter. No sudden death was reported during 3144 SUDC sibling life-years (eFigure in the Supplement).

### Analysis of 391 Interviews of Sudden Child Death Aged 1 to 6 Years 

Among the 622 families, 414 were interviewed; of these, 94.4% (n = 391; 127 SEDC and 264 SUDC) decedents were aged 1 to 6 years (Figure). Since only 21 children were older than 7 years, the small sample and wide age range limited our power to explore risk factors; thus, we focused our analyses only on children aged 1 to 6 years. Among the 622 total and 391 interviewed children aged 1 to 6 years, there was the same proportion of SEDC (208 [33.4%]) and SUDC (414 [66.6%]) cases. Frequencies reported reflect available information, as some questions were not answered by every interviewee.

Detailed demographic and interview histories for the SEDC and SUDC are presented in eTable 2 in the Supplement. Of these 391 interviews, 59.1% (n = 231) of the decedents were male, the mean (SD) age was 24.9 (12.8) months, and 26.6% (n = 104) had FS. The reported races of decedents included 83.1% white (325 of 391), 9.0% mixed or other (35 of 391), and 3.1% black or African American (12 of 391).

### SEDC Deaths

Among the SEDC, 115 (90.6%) were certified as natural deaths, 73 (63.5%) were attributed to infection (mainly pneumonia and viral), and 12 (9.4%) as accidental deaths (mainly accidental asphyxia) (eTable 3 in the Supplement). There were no homicides or suicides as these were exclusionary. The median (IQR) age at SEDC was 22 (17-31) months. Most were male (85 [66.3%]) and white (104 [81.9%]).

Cases of FS were reported in 28 SEDC (22.1%; 95% CI, 14.8%-29.3%); 17 (58.6%) had simple FS, 6 (20.7%) had complex FS, and 5 (17.2%) had both. There was 1 (0.8%) case each of afebrile seizure, syncope, and sleep apnea (eTable 2 in the Supplement). Although 13 SEDC (10.2%) were attributed to seizures (10 FS, 1 afebrile seizure, 1 unknown seizure type, and 1 without known seizure history), an additional 5 (4%) had a history of seizures (4 FS, 1 afebrile seizure) not associated with death by the medical examiner or coroner of record. Family FS history was reported in 24 of 117 cases (20.5%), afebrile seizures in 31 of 125 cases (24.8%), syncope in 21 of 113 cases (18.6%), and sudden unexplained deaths, including SIDS, in 7 of 115 cases (6.1%). Both decedent and family history of FS were reported in 11 of 117 cases (9.4%) and decedent and/or family history of FS in 38 of 117 cases (32.5%).

Among SEDC, 49 of 120 cases (40.8%) reported a terminal fever, 111 of 127 cases (87.4%) occurred during a sleep period, 60 of 94 cases (63.8%) were found in a crib, 8 of 87 cases (9.2%) were sharing a sleep surface, 74 of 93 (79.6%) were discovered prone, and 44 of 84 cases (52.4%) were found face down.

### SUDC Deaths

The median (IQR) age for SUDC was 20 (16-27) months. Most were male (146 [55.3%]), white (221 of 262 [84.4%]), and born full-term (111 of 136 [81.6%]). The child’s development was described as normal in 201 cases (76.1%). Vaccinations were up to date in 214 cases (81.1%), with 130 of 150 (86.7%) receiving their last vaccination more than 2 weeks before death.

The mother’s median (IQR) age at decedent’s birth was 30 (27-34) years. Family medical history identified FS in 87 of 241 cases (36.1%), afebrile seizures in 73 of 243 cases (30.0%), syncope in 40 of 231 cases (17.3%), and sudden unexplained deaths, including SIDS, in 10 of 240 cases (4.2%). Forty-one of 241 cases (17.0%) reported both a case and family history of FS, and half (122/241 [50.6%]) reported a case and/or family history of FS.

Of the 76 SUDC cases with FS (28.8%; 95% CI, 23.3%-34.2%), 62 (81.6%) had simple FS, 6 (7.9%) had complex FS, and 8 (10.5%) had both. Six of 59 (10.2%) had a witnessed seizure within 48 hours of death, and another 4 (6.8%) witnessed 1 between 48 hours and 1 month of death. Seven SUDC cases reported afebrile seizures, 1 of whom had a history of simple FS. The median (IQR) age of the first FS was 15 (12-18) months and first afebrile seizure was 12 (9-14) months.

Nearly all children with SUDC (253 [95.8%]) were found unresponsive during a sleep period; 173 of 211 cases (82%) were discovered prone and 122 of 192 cases (63.5%) were found face down. Most (135 of 226 [59.7%]) slept in a crib. In 16 of 210 cases (7.6%), the child shared a sleep surface. In 112 of 245 cases (45.7%), a terminal fever was reported.

### Potential Risk Factors for SUDC vs SEDC

Febrile seizures were reported in 76 of 264 cases (28.8%) of SUDC and 28 of 127 cases (22.1%) of SEDC (adjusted *P* > .05) (eTable 2 in the Supplement). Cases of SUDC with FS had higher rates of hospitalization and family history of FS than other SEDC or SUDC groups (Table 1). Deaths during apparent sleep were more common among SUDC vs SEDC cases (OR, 4.61; 95% CI, 1.92-11.09; adjusted *P* = .008) (Table 1). The SEDC and SUDC groups were similar in all other interview factors including parental ages at decedent’s birth; prior pregnancies and miscarriages; gestational term deliveries; delivery complications; rates of breastfeeding; normal child development; receiving early intervention; surgical, medical, and allergy histories; up-to-date vaccinations (78% and 81.1%); time between last vaccinations and death; smoke exposure; day of the week and season of death; terminal fever; position found; sleep surface for sleep-associated deaths; bed sharing; witnessed death; or family histories of syncope, afebrile seizures, or sudden unexplained deaths (eTable 2 and eTable 4 in the Supplement)

**Table 1.** Potential Risk Factors in 391 Sudden Pediatric Deaths

Factors	No. (%)				Adjusted *P* Value^a^^,^^b^
	Total SEDC Deaths (n = 127)	SUDC With FS (n = 76)	SUDC Without Seizure History (n = 181)	Total SUDC Deaths (n = 264)	
Hospitalization history	48 (37.8)	50 (65.8)^b^	53 (29.3)^b^	107 (40.5)	<.001^b^
Apparent death during sleep	111 (87.4)^c^	75 (98.7)	174 (96.1)	256 (97.0)^c^	.008^c^
First- and second-degree family history of FS, No./No. (%)^d^	24/117 (20.5)	41/76 (54.0)^e^	45/160 (28.1)^e^	87/241 (36.1)	.003^e^

Abbreviations: FS, febrile seizures; SEDC, sudden explained death in childhood; SUDC, sudden unexplained death in childhood.

^a^ For all significant and not calculable adjusted *P* values, see eTable 4 in the Supplement.

^b^ Significant adjusted *P* value of less than .001 for hospitalization history between SUDC with FS and SUDC without seizure history.

^c^ Significant adjusted *P* value of .008 for apparent death during sleep between total SEDC deaths and total SUDC deaths.

^d^ No./No. denotes sample size/population size.

^e^ Significant adjusted *P* value of .003 for first- and second-degree family history of FS between SUDC with FS and SUDC without seizure history.

### SUDC With FS vs SUDC Deaths Without Seizure History

Compared with SUDC cases without a seizure history, those with FS were older (median, 25 months; IQR, 19-36 vs median, 18 months; IQR, 15-25; adjusted *P* = .003), with greater family FS history (41 of 76 [54.0%] vs 45 of 160 [28.1%]; OR, 2.99; 95% CI, 1.70-5.28; adjusted *P* = .003), and higher rates of prior hospitalizations (50 [65.8%] vs 53 [29.3%]; OR, 4.64; 95% CI, 2.62-8.23; adjusted *P* < .001) (Table 1). Ages at explained natural deaths were older than SUDC cases without a seizure (median, 23 months; IQR, 18-31 vs median, 18 months; IQR, 15-25; adjusted *P* = .04).

### Risk Factors Associated With FS by Explained Natural Deaths and SUDC Deaths

Natural deaths with simple FS had higher hospitalization rates than natural deaths without seizure history (12 [70.6%] vs 23 [26.4%]; OR, 6.68; 95% CI, 2.12-21.03; adjusted *P* = .006). Cases of SUDC with simple FS were more likely to have family history of afebrile seizures (26 of 61 [42.6%] vs 34 of 164 [20.7%]; OR, 2.84; 95% CI, 1.51-5.35; adjusted *P* = .02) and FS (31 of 62 [50.0%] vs 45 of 160 [28.1%]; OR, 2.56; 95% CI, 1.39-4.68; adjusted *P* = .04) than SUDC cases without seizure history (Table 2). No other significant differences were identified from interview histories.

**Table 2.** Potential Risk Factors in 371 FS-Associated Deaths

Factor	Explained Natural Deaths			SUDC			Adjusted *P* Value^a^
	Simple FS Only (n = 17)	Simple Plus Complex or Complex FS Only (n = 10)	No Seizure History (n = 87)	Simple FS Only (n = 62)	Simple Plus Complex or Complex FS Only (n = 14)	No Seizure History (n = 181)	
Hospitalization history, No. (%)	12 (70.6)^b^	9 (90.0)	23 (26.4)^b^	41 (66.1)^c^	9 (64.3)	53 (29.3)^c^	.006^b^ and <.001^c^
First- and second-degree family history, No./No. (%)^d^							
FS	7/17 (41.2)	5/10 (50.0)	14/78 (18.0)	31/62 (50.0)^e^	10/14 (71.4)	45/160 (28.1)^e^	.04^e^
Afebrile seizures	8/17 (47.1)	3/10 (30.0)	21/80 (26.3)	26/61 (42.6)^f^	6/13 (46.2)	34/164 (20.7)^f^	.02^f^

Abbreviations: FS, febrile seizures; SUDC, sudden unexplained death in childhood.

^a^ Adjusted *P* values using the Holm-Bonferroni Method.

^b^ Significant adjusted *P* value of .006 for hospitalization history between SEDC natural, simple FS only and SEDC natural, and no seizure history.

^c^ Significant adjusted *P* value of less than .001 for hospitalization history between SUDC, simple FS only and SUDC, and no seizure history.

^d^ No./No. denotes sample size/population size.

^e^ Significant adjusted *P* value of .003 for first- and second-degree family history of FS between SUDC, simple FS only and SUDC, and no seizure history.

^f^ Significant adjusted *P* value of .003 for first- and second-degree family history of afebrile seizures between SUDC, simple FS only and SUDC, and no seizure history.

## Discussion

Among 391 consecutively collected interviews of family members of children aged 1 to 6 years at SUDC, we found elevated rates of FS among both SEDC (22.1%) and SUDC (28.8%), compared with population norms (2%-5%).^8,9^ Among the original 622 cases of SUDC and SEDC combined, with some families followed for 15 years, we identified no SUDC death across 4236 sibling life-years, as of October 24, 2018, including 3144 SUDC sibling life-years (eFigure in the Supplement). We also reported, to our knowledge, the most comprehensive data on maternal history, child and family medical history, and circumstances of death in SUDC and SEDC in young children.

Our study, to our knowledge, for the first time, identified a high prevalence of FS in cases certified as SEDC. Prior studies and ours found a high prevalence of FS in cases certified as SUDC.^1,10,14^ Compared with the white population (mean FS incidence, 3.5%; range, 2%-5%),^8,9^ the predominant race in our cohort, the FS rate was approximately 8-fold higher among SUDC cases and approximately 6-fold higher among SEDC cases. Our findings implicate FS as a contributing factor or the cause of death via a SUDEP-like process in some SUDC cases.^12^ A similar seizure-induced mechanism likely contributes to some SEDC, but the death is attributed to another cause (eg, infection). In addition to the epidemiologic data across numerous series,^1,10,14^ the pathogenic link between FS SUDC and SUDEP is supported by a child aged 20 months whose death was recorded during video electroencephalogram monitoring and occurred immediately following febrile status epilepticus. The electroencephalogram revealed cerebral suppression followed by bradycardia prior to death, identical to video electroencephalogram recordings in SUDEP cases.^15,16^

We speculate that FS occur in some children without a known FS history but are never recognized by parents or caregivers. Further, seizures in young children only may cause apnea or other nonmotor symptoms that go unrecognized or are subclinical.^17^ A similar ictal pattern—isolated apnea—could result from illness or fever in young children predisposed to FS, but it may remain unknown unless lethal. Postmortem evidence of terminal seizures is often absent or nonspecific, ie, a negative autopsy.^18^ Explained deaths attributed to asphyxia could include FS cases where the child remained prone owing to the postictal state. Common stigmata of convulsive seizures in older children and adults, such as tongue bite or urinary incontinence, are either infrequent (tongue bite) or difficult to identify (urinary incontinence) in young children. A longitudinal, population-based study in Denmark concluded long-term mortality is not increased in children with simple FS—the most frequent seizure in our series (in 81.6% of SUDC cases and 60.7% of SEDC cases).^19^ However, a recent Danish population-based study found a nearly 2-fold increase in sudden cardiac deaths among individuals aged 1 to 30 years with an FS history, compared with living controls and sudden deaths owing to motor vehicle crashes. This could possibly be associated with ion channel genes expressed in both brain and heart or misattribution of cardiac death instead of seizure-associated cause of death.^20^

Our study supports prior observations and provides novel SUDC insights. We confirmed associations of SUDC in males, full-term gestation, predominantly normal development, FS family history, death during a sleep period, and being discovered unresponsive in prone position. Sudden unexplained death in childhood occurred at approximately 20 months of age (older in cases with FS vs those without). The prone position, a SIDS risk factor, increases asphyxia risk and decreases stability of the autonomic nervous system by decreasing sympathetic tone and heart rate variability.^21,22^ Similarly, SUDEP cases are often discovered prone, especially after tonic-clonic seizures.^23,24^

Our data detail the profile of SUDC and SEDC primarily among children aged 1 to 6 years. As in SUDC, we also found a higher proportion of boys among explained deaths compared with US population norms.^1,7,11,25^ Full-term gestation rate for both groups (81.6% and 84.7%) fell below the national average of 90.2%.^26^ Sudden unexplained death in childhood and SEDC maternal age was older at decedent’s birth (median, 30 years; IQR, 27-34 and median, 31 years; IQR, 28.5-33) compared with SIDS mothers (76% were <29 years).^26^ Bed sharing, another risk factor for SIDS, was infrequent (7.6% and 9.2%). Strikingly similar prevalence rates for SUDC and SEDC factors were measured, except for a 4.5-fold increased rate of apparent death during sleep among SUDC. No other factors significantly differed between SUDC and SEDC.

We found no increase in sudden cardiac death risk factors in our study.^27^ While rare variants associated with inherited cardiac conditions occur in 14% of SIDS cases,^28^ genetic analyses of SUDC victims are limited but established that cardiac channelopathies likely cause some of the deaths that often occur in sleep.^29^ The prevalence of cardiac and other genetics risks in SUDC requires additional studies. We found similar rates of family history of sudden deaths among SUDC and SEDC (4.2% and 6.1%).

Both SIDS and SUDC are arbitrarily distinguished by 1 day of life, yet SIDS has received public efforts to decrease its incidence, while SUDC has not. The Safe Sleep and the Back to Sleep campaigns contributed to the approximately 54% decrease in SIDS since the early 1990s,^4^ while SUDC among children aged 1 to 4 years increased slightly (1.3 to 1.5 per 100 000 crude death rate from 1999-2017).^5^ In the last 15 years, the SUDC rate doubled in Ireland.^30^ We need accurate SUDC surveillance to gain more insights into its mechanisms and risk factors.

### Limitations

Limitations of this study include interview data collected at variable intervals after the child’s death, which could introduce recall bias, and a lack of medical records or autopsy report reviews. Our series was not population-based but from families enrolled in the SUDC Foundation and completed an interview, creating potential referral biases. Medical histories, including the assessment of developmental norms and vaccination rates were collected from parents and not medical records. Although vaccination rates were lower than expected in our series (SEDC 78%, SUDC 81.1%), methodology differences prevent reliable comparisons with national statistics.^31^ Our study was biased to more white and fewer black individuals than the US population. For 2017, the Centers for Disease Control and Prevention reported that 63.8% SUDC deaths in children aged 1 to 4 years were white and 30.9% were black with SUDC death rates in this age group being 2.1-fold higher among black individuals than whites individuals (2.7 deaths per 100 000 vs 1.3 deaths per 100 000).^5^ This suggests that our series cannot address other risk factors underlying this disparity. Systemic, population-based assessments are needed to address this limitation.

## Conclusions

To our knowledge, our study is the largest published series of SUDC and SEDC cases to date, with 391 probands and in-depth family interviews of children aged 1 to 6 years with sudden deaths. The largest prior SUDC series examined 151 cases and identified risk factors including a personal or family history of FS, death during sleep, and mild hippocampal malformations.^11^ We identified significant elevated rates of FS among both SUDC and SEDC, suggesting a SUDEP-like mechanism could contribute to some sudden deaths in young children.^8,9^ However, most cases had no history of seizures. Although this does not exclude the possibility of unwitnessed or unrecognized seizure activity, it supports that multiple unrecognized causes that contribute to these deaths remain unexplained. Sudden unexplained death in childhood only differed from SEDC in having a higher prevalence of death during apparent sleep. Parents can be reassured that the risk to siblings for sudden death is low.

Sudden unexplained death in childhood remains a major public health concern that has received little clinical, epidemiological, and genetic research. The National Institutes of Health and Centers for Disease Control and Prevention sudden death in the young registry studies the incidence, causes, and risk factors for sudden deaths in children (ages 0-19 years) in 13 states or jurisdictions and is composed of 75% infants with limited children aged 1 to 6 years with sudden deaths.^32^ Sudden unexplained death in childhood incidence is underestimated owing to inaccurate and inconsistent death certification practices and a bias to find a cause of death.^6^ Current autopsy practices are constrained by resources and failure to identify functional causes of death associated with a negative autopsy finding, including cardiac arrhythmias and seizures.

To understand and prevent sudden child deaths, SUDC and SEDC require population-based studies using consistent death scene investigations, comprehensive and standardized autopsy methods (eg, whole brain and heart examinations) with uniform certification practices, infectious, metabolic, and genetic investigations and in-depth family interviews to correlate findings.^1,7,11^ To develop and assess preventive strategies for deaths associated with FS, we need population-based studies to further define the epidemiology and risk factors and identify biomarkers of patients with FS who are at high risk of SUDC.

## Notes

Supplement. **eTable 1.** SUDC Foundation Family Interview Data Collected **eTable 2.** Detailed Demographic and Interview Histories for the SEDC and SUDC (N = 391) **eFigure.** Sibling Age Distributions With Known Birthdate as of October 24, 2018, in Years (n = 473) **eTable 3.** Cause of Death for Sudden, Unexpected, and Explained Cases (SEDC) With Interviews (N = 127) **eTable 4.** SEDC vs SUDC Interview Factors With Adjusted *P* Values ≠1.000
