# Error in Figure

**DOI:** 10.1001/jamanetworkopen.2019.9796

**Published:** 2019-07-31

In the Original Investigation titled “Potential Role of Febrile Seizures and Other Risk Factors Associated With Sudden Deaths in Children,”^1^ published April 26, 2019, there was an error in the Figure. The text reading “181 SUDC with seizure history” should have read “181 SUDC without seizure history.” This article has been corrected.^1^
