# Supplementary material for: Potential Role of Febrile Seizures and Other Risk Factors Associated With Sudden Deaths in Children
Source: JAMA Netw Open. 2019 Apr 26;2(4):e192739. doi: 10.1001/jamanetworkopen.2019.2739 (PMC6487567; doi:10.1001/jamanetworkopen.2019.2739)
Supplement: Supplement. — eTable 1. SUDC Foundation Family Interview Data Collected eTable 2. Detailed Demographic and Interview Histories for the SEDC and SUDC (N = 391) eFigure. Sibling Age Distributions With Known Birthdate as of October 24, 2018, in Years (n = 473) eTable 3. Cause of Death for Sudden, Unexpected, and Explained Cases (SEDC) With Interviews (N = 127) eTable 4. SEDC vs SUDC Interview Factors With Adjusted P Values ≠1.000 [file jamanetwopen-2-e192739-s001.pdf]

Supplementary Online Content

Crandall LG, Lee JH, Stainman R, Friedman D, Devinsky O. Potential role of febrile seizures and other risk factors associated with sudden deaths in children. *JAMA Netw Open*. 2019;2(4):e192739. doi:10.1001/jamanetworkopen.2019.2739

eTable 1. SUDC Foundation Family Interview Data Collected

eTable 2. Detailed Demographic and Interview Histories for the SEDC and SUDC (N = 391)

eFigure. Sibling Age Distributions With Known Birthdate as of October 24, 2018, in Years (n = 473)

eTable 3. Cause of Death for Sudden, Unexpected, and Explained Cases (SEDC) With Interviews (N = 127)

eTable 4. SEDC vs SUDC Interview Factors With Adjusted *P* Values ≠1.000

This supplementary material has been provided by the authors to give readers additional information about their work.

© 2019 Crandall LG et al. *JAMA Network Open*.

eTable 1. SUDC Foundation Family Interview Data Collected

**Demographics**	• Gender<br>• Race and ethnicity<br>• Child's DOB and DOD<br>• Mother's age at time of birth and death<br>• Father's age at time of birth and death
**Maternal History**	• Pregnancy history (# para, # gravida, # total live births, # miscarriages, # abortions, # still births)<br>• Order of pregnancy of deceased?<br>• How long did it take for the mom to get pregnant?<br>• Was assisted reproductive technology (fertility medicals, IVF, surrogacy) used to conceive the child?<br>• Any medication and drug use during pregnancy?
**Birth History and Infant History**	• Was the decedent born full-term?<br>• Any complications with delivery?<br>o Any history of pre-eclampsia<br>• Did they sleep supine?<br>• Did they use a pacifier?<br>• Was the infant breastfed? If so, for how long?<br>• Did the infant ever experience blue spells, paleness, or limpness?<br>• Was the child exposed to smoke? Did anyone smoke in home? Did mom smoke during pregnancy?
**Medical History**	• In their life, did they have history of fasting more than 12 hrs or a history of vomiting more than 12 hours?<br>• Did they have a history of sleep apnea, chronic lung disease, asthma, GERD, muscle weakness, congenital heart disease, irregular heartbeats, choking spells, bradycardia, or syncope?<br>• Did they have any allergies? If so, what were they?

© 2019 Crandall LG et al. *JAMA Network Open*.

	• Did they child have any seizures? Were they febrile? Were they simple or complex febrile seizures?<br>• Did they have any surgeries?<br>• Were they ever hospitalized?<br>• Were their vaccinations up to date? When was their last vaccination?<br>• Did they take any medications on a regular basis?
**Developmental and Social Factors**	• How was their development?<br>• Do you or your doctor have any concerns regarding your child's development?<br>• Did they have any special needs or receive any special services?<br>• What were their childcare arrangements?<br>• Did they go to daycare? If so, from what age?<br>• If they were school aged, what grade were they in and how were they performing in school?
**Family History of First and/or Second Degree Relatives**	• Febrile seizures, epilepsy or seizures, psychiatric disorders, autism, multiple sclerosis, migraines?<br>• Apparent life-threatening event (ALTE), unexplained death less than 50 years of age, sudden explained death of any age?<br>• Sleep Apnea, Sleep Disorders?<br>• Premature births?<br>• Cardiac Disease, Crohn's Disease, Lupus, Rheumatoid Arthritis, Diabetes Mellitus Types 1 and 2, Tourette's syndrome, Autoimmune Disorders.<br>• Any other family medical history you think is worth mentioning?

© 2019 Crandall LG et al. *JAMA Network Open*.

**Circumstances of Child's Death**	• Was there any recent head trauma within the last two weeks of their life?<br>• What were their terminal symptoms in the last 48 hours?<br>o Was there a fever?<br>• What medications did they take in the last 24 hours?<br>• When and where was the child last known alive and by whom?<br>• When and where was the child found responsive and by whom?<br>o What day of the week did it occur?<br>o What season was it?<br>• Was the death witnessed?<br>• Was it a sleep-related death?<br>o Was the sleep surface?<br>o Please describe the sleep environment.<br>o Was the child sharing the sleep surface with another human being or animal?<br>• What were the body, face, and extremities positions when the child was found?<br>• Was the nose or mouth obstructed?<br>• Were there any fluids around the face?<br>• What devices were operating in the room?
**Death Investigations**	• Describe the investigation performed<br>o Was there a scene investigation?<br>o Was there an autopsy?<br>o Were there any interviews? If so, who conducted the interviews and by what office?<br>• What was the final cause of death certification?

© 2019 Crandall LG et al. *JAMA Network Open*.

eTable 2. Detailed Demographic and Interview Histories for the SEDC and SUDC (N = 391)

Factors	Total SEDC Deaths (N=127)	Total SUDC Deaths (N=264)
**Demographics**		
Median Age (mo) at death (IQR)	22 (17-31)	20 (16-27.25)
Males	85 (66.3%)	146 (55.3%)
Race and Ethnicity†		
White	104/127 (81.9%)	221/262 (84.4%)
Black/African American	4/127 (3.2%)	8/262 (3.1%)
Asian/Pacific Islander	4/127 (3.2%)	5/262 (1.9%)
Hispanic	3/127 (2.4%)	5/262 (1.9%)
Mixed/Other	12/127 (9.5%)	23/262 (8.8%)
Median mother's age at time of birth (IQR)	31 (28.5-33)	30 (27-34)
Median father's age at time of birth (IQR)	32.5 (29.75-36)	32.5 (29-36.25)
**Maternal and Birth History****		
Median para (IQR)	2 (2-3)	2 (1-3)
Mother had miscarriage†	26/88 (29.6%)	63/186 (33.9%)
Median number of miscarriages	0 (0-1)	0 (0-1)
Pregnancies lost/Total Pregnancies	13.7%	23.2%
Birth order of deceased (IQR)	2 (1-3)	2 (1-3)
NICU stay	10 (7.9%)	15 (5.7%)
Normal Delivery/No Complications	53 (41.7%)	101 (38.3%)
Term †	61/72 (84.7%)	111/136 (81.6%)
**Child Medical History and Social Factors**		
Normal Child Development	95 (74.8%)	201 (76.1%)
Received early intervention	7 (5.5%)	25 (9.5%)
Allergy history	29 (22.8%)	33 (12.5%)
Hospitalization history	48 (37.8%)	107 (40.5%)
Surgical history	16 (12.6%)	23 (8.7%)
FS history	28 (22.1%)	76 (28.8%)

© 2019 Crandall LG et al. *JAMA Network Open*.

Factors	Total SEDC Deaths (N=127)	Total SUDC Deaths (N=264)
Afebrile seizures history	1 (0.8%)	7(2.7%)ƚ
Syncope history	1 (0.8%)	7 (2.7%)
Sleep apnea history	1 (0.8%)	3 (1.1%)
Breastfed < 3 months†	20/98 (20.4%)	51/203 (25.1%)
Breastfed > 3 months†	62/98 (63.3%)	108/203 (53.2%)
Vaccinations up to date at death	99 (78.0%)	214 (81.1%)
Last vaccination: < 2 weeks prior to death†	6/65 (9.2%)	20/150 (13.3%)
Prenatal smoke exposure†	1/94 (1.1%)	3/186 (1.6%)
Postnatal secondhand smoke†	7/94 (7.5%)	23/186 (12.4%)
**Factors Related to Child FS History**		
FS History	28/127 (22.1%)	76 (28.8%)
Afebrile Seizure	1 (0.8%)	7 (2.7%)ƚ
Median age (mo) at first Sz (FS/aFS) (IQR)	16 (12-20);N=29	14 (11-18);N=79
Median duration (min) of the longest Sz (FS/aFS) (IQR)	2.75 (1-3)	3 (1-5)
Last Sz: < 48 h prior to death†	2/21 (9.5%)	7/61 (11.5%)
Last Sz: btwn 48 h and 1 mo prior to death†	0/21 (0.0%)	4/61 (6.6%)
Last Sz: > 1 month prior to death†	19/21 (90.5%)	50/61 (82.0%)
**Family History*†**		
FS	24/117 (20.5%)	87/241 (36.1%)
First Degree Relatives	21/117 (18.0%)	57/241 (23.7%)
Second Degree Relatives	10/117 (8.6%)	45/241 (18.7%)
Afebrile seizures	31/125 (24.8%)	73/243 (30.0%)
Syncope	21/113 (18.6%)	40/231 (17.3%)
Sudden unexplained deaths	7/115 (6.1%)	10/240 (4.2%)
**Circumstances of Death**		
Day of Death = Weekday	68 (53.5%)	141 (53.4%)
Season of death		
Fall	28 (22.1%)	53 (20.1%)
Spring	20 (15.8%)	57 (21.6%)

© 2019 Crandall LG et al. *JAMA Network Open*.

Factors	Total SEDC Deaths (N=127)	Total SUDC Deaths (N=264)
Summer	26 (20.5%)	44 (16.7%)
Winter	53 (41.7%)	110 (41.7%)
Terminal fever 48H before death†	49/120 (40.8%)	112/245 (45.7%)
**Circumstances of Death for Sleep-Related Child Deaths Only**		
Apparent death during sleep	111 (87.4%)	256 (97.0%)
Body position prone at death†	74/93 (79.6%)	173/211 (82.0%)
Face down at death†	44/84 (52.4%)	122/192 (63.5%)
Surface Discovered Unresponsive†		
Crib	60/94 (63.8%)	135/226 (59.7%)
Bed/Mattress (Toddler, Twin, Full, Queen, King)	26/94 (27.7%)	61/226 (2%)
Other Surfaces (Couch, chair, floor, playpen/playyard, car seat)	8/94 (8.5%)	30/226 (13.3%)
Sleeping conditions at death˚		
Pillow	37 (29.1%)	76 (28.8%)
Blanket/Quilt	56 (44.1%)	127 (48.1%)
Stuffed Toy	38 (29.9%)	76 (28.8%)
Sleeping with others	8/87 (9.2%)	16/210 (7.6%)
Death Witnessed†	13/127 (10.2%)	16/262 (6.1%)

Unless otherwise noted, each cell is in the following format: n/N([{n/N}*100])% with n being the sample size and N being population size of each column.
FS: Febrile Seizure; aFS: Afebrile Seizure
ł One subject also had a history of simple FS
†: No./Total No. with known data (%)
†† Unable to calculate p-value due to non-exclusive answers
˚: Subjects may fall under multiple categories, cannot conduct statistical analysis
**: Subjects answered to birth complications in a narrative form; counts cannot be added toward the total, thus unable to calculate p-value
x/n= the number of cases divided by total number of cases in this subgroup with any Sz Hz

Significant Adjusted p-values are listed in Table 1

© 2019 Crandall LG et al. *JAMA Network Open*.

eFigure. Sibling Age Distributions With Known Birthdate as of October 24, 2018, in Years (n = 473)

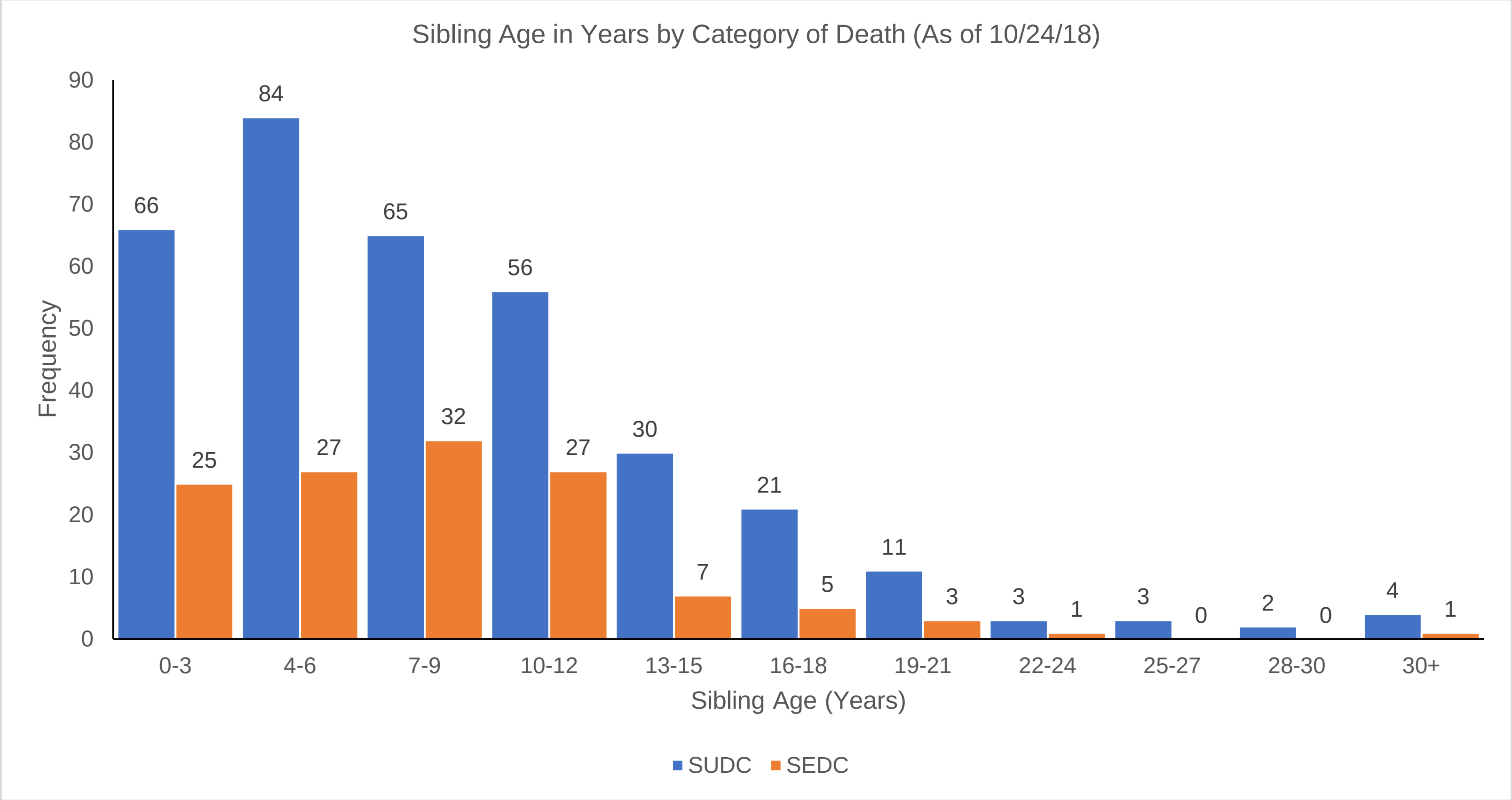

© 2019 Crandall LG et al. *JAMA Network Open*.

eTable 3. Cause of Death for Sudden, Unexpected, and Explained Cases (SEDC) With Interviews (N = 127)

**Final Diagnosis**	**Count**
Accidental Deaths	***N=12***
Asphyxia Related	10
Drowning	1
Hyperthermia	1
Natural Infectious Deaths	***N=73***
Pneumonia	22
Viral Infection	17
Respiratory Infection (Bronchitis, Bronchiolitis, Tracheitis, Laryngitis)	10
Myocarditis	7
Influenza	5
Bacterial Infection	4
Meningitis	2
Encephalitis	2
Sepsis	2
Unspecified Infection	2
Natural Non-Infectious Deaths	***N=42***
Seizure-Related Deaths	13
Cardiomyopathy	8
Cardiac Dysrhythmia	6
Asthma	3
Congenital Heart Defects	3
Acute Congestive Heart Failure	1
Anoxic Brain Injury	1
Cerebral Cortical Dysplasia	1
Meckells Diverticulum	1
Hyperglycemia	1
Congenital Metabolic Disorder	1
Dehydration	1
Disseminated Intravascular Coagulation	1
AV Nodal Artery Dysplasia	1

© 2019 Crandall LG et al. *JAMA Network Open*.

eTable 4. SEDC vs SUDC Interview Factors With Adjusted *P* Values ≠1.000

**Factors**	**Total SEDC Deaths Vs. Total SUDC Deaths Adjusted p-Values***
Gender	0.9423
Race and Ethnicity	NC
Smoke History	NC
Child History	
Allergy history	0.2990
Afebrile Sz	NC
Syncope	NC
Sleep apnea	NC
Family History	
FS	0.0974
Death during sleep	0.0080†

*Adjusted P-Values using the Holm-Bonferroni Method; NC = not calculable, expected values < 5 so unable to perform chi-square tests.
† Denotes p-value <.05

© 2019 Crandall LG et al. *JAMA Network Open*.
